# Initial treatment response and short-term mortality of spontaneous bacterial peritonitis in cirrhotic patients with hepatocellular carcinoma

**DOI:** 10.1038/s41598-023-32006-8

**Published:** 2023-04-13

**Authors:** Chang Hun Lee, Hye Jin Kang, Song Yi Yu, Seung Young Seo, Seong Hun Kim, Sang Wook Kim, Seung Ok Lee, Soo Teik Lee, In Hee Kim

**Affiliations:** Department of Internal Medicine, Research Institute of Clinical Medicine of Jeonbuk National University-Biomedical Research Institute of Jeonbuk National University Hospital, Jeonbuk National University Medical School and Hospital, Geonjiro 20, Dukjin-Gu, Jeonju, Jeonbuk 54907 South Korea

**Keywords:** Gastroenterology, Hepatology

## Abstract

This study aimed to investigate the initial treatment response and short-term mortality of spontaneous bacterial peritonitis (SBP) in cirrhotic patients with hepatocellular carcinoma (HCC) compared with those without HCC. A total of 245 patients with liver cirrhosis diagnosed with SBP between January 2004 and December 2020 were included. Of these, 107 (43.7%) were diagnosed with HCC. Overall, the rates of initial treatment failure, 7-day and 30-day mortality were 91 (37.1%), 42 (17.1%), and 89 (36.3%), respectively. While the baseline CTP score, MELD score, culture-positive rate, and rates of antibiotic resistance did not differ between both groups, patients with HCC had a higher rate of initial treatment failure than those without HCC patients (52.3% vs. 25.4%, *P *< 0.001). Similarly, 30-day mortality was also significantly higher in patients with HCC (53.3% vs. 23.2%, *P *< 0.001). In the multivariate analysis, HCC, renal impairment, CTP grade C, and antibiotic resistance were independent factors for initial treatment failure. Furthermore, HCC, hepatic encephalopathy, MELD score, and initial treatment failure were independent risk factors for 30-day mortality, with statistically significant poor survival outcomes in patients with HCC (*P *< 0.001). In conclusion, HCC is an independent risk factor for initial treatment failure and high short-term mortality in patients with cirrhosis with SBP. It has been suggested that more attentive therapeutic strategies are required to improve the prognosis of patients with HCC and SBP.

## Introduction

Spontaneous bacterial peritonitis (SBP) is a spontaneous infection of ascitic fluid without an evident intra-abdominal surgically treatable source of infection^1,2^. SBP is a common, recurrent complication of cirrhosis associated with poor outcome^3–5^. The prevalence of SBP in hospitalized cirrhotic patients with ascites is approximately 10–30%^6^. Bacterial infection precipitates an excessive inflammatory responses accompanied by hemodynamic dysfunction in cirrhosis which can lead to serious complications, such as shock, liver failure, and death^7^. Thus, although the mortality of SBP has been reduced to approximately 10–50% with early recognition and antibiotic therapy, it still remains a major cause of mortality in patients with cirrhosis^7^.

Patients with cirrhosis have an increased risk of developing hepatocellular carcinoma (HCC), and the 5-year cumulative incidence of HCC ranges from 4 to 30% depending on the etiology of cirrhosis^8^. In addition, the prevalence of HCC in cirrhotic patients with SBP is known to be high at approximately 20%^9^. There are a few studies that have evaluated the prognosis in patients with SBP accompanied by HCC^10–14^. However, data evaluating the impact of HCC on initial treatment outcomes and short-term mortality in patients with cirrhosis with complicated SBP are still limited.

In this study, we aimed to compare the clinical features including initial treatment responses and short-term mortality of SBP among cirrhotic patients with HCC versus those without HCC. We also analyzed factors associated with initial treatment failure and short-term mortality.

## Results

### Clinical characteristics of SBP between HCC patients versus non-HCC patients

The study included 245 cases of SBP diagnosed in patients with liver cirrhosis between January 2004 and December 2020. Baseline characteristics of the enrolled patients at SBP diagnosis are shown in Table 1. Of the 245 subjects evaluated, the mean age was 58.9 years and males were predominant (80.8%). The etiology of liver cirrhosis was alcoholic in 85 (34.7%) patients. Seventy-four patients (30.2%) were prescribed antibiotics within 60 days, and 143 patients (58.4%) were diagnosed with community-acquired SBP. The most common presenting manifestations were abdominal pain (71.8%), fever (42.0%), and hepatic encephalopathy (20.0%). Forty-one patients (16.7%) had concomitant other site infections, including pneumonia, urinary tract infections, and bacteremia. Classification according to the Child–Pugh scoring system showed that 25.7% of the patients were Child B and 74.3% Child C. The mean MELD score was 19.6 ± 9.2. At the time of SBP diagnosis, 107 (43.7%) patients had HCC.

**Table 1:** Clinical characteristics of patients.

Characteristics	Non-HCC patients (n = 138)	HCC patients (n = 107)	Total (n = 245)	*P* value
Age, years	57.6 ± 12.0	60.5 ± 8.6	58.9 ± 10.7	0.029
Male sex	107 (77.5%)	91 (85.0%)	198 (80.8%)	0.188
Etiology of cirrhosis, alcohol (vs. non-alcohol)	58 (42.0%)	27 (25.2%)	85 (34.7%)	0.009
Diabetes mellitus	34 (24.6%)	21 (19.6%)	55 (22.4%)	0.437
Acqusition of SBP, CA (vs. NC)	87 (63.0%)	56 (52.3%)	143 (58.4%)	0.120
Concomitant other site infections	24 (17.4%)	17 (15.9%)	41 (16.7%)	0.889
Pneumonia	9 (6.5%)	7 (6.5%)	16 (6.5%)	1.000
UTI	6 (4.3%)	1 (0.9%)	7 (2.9%)	0.229
Bacteremia	9 (6.5%)	10 (9.3%)	19 (7.8%)	0.563
Previous use of antibiotics within 60 days	26 (18.8%)	48 (44.9%)	74 (30.2%)	< .001
NSBB administration	61 (44.2%)	34 (31.8%)	95 (38.8%)	0.065
Clinical manifestations				
Fever	59 (42.8%)	44 (41.1%)	103 (42.0%)	0.900
Abdominal pain	97 (70.3%)	79 (73.8%)	176 (71.8%)	0.640
Diarrhea	22 (15.9%)	15 (14.0%)	37 (15.1%)	0.813
Hepatic encephalopathy	24 (17.4%)	25 (23.3%)	49 (20.0%)	0.310
GI hemorrhage	21 (15.2%)	12 (11.2%)	33 (13.5%)	0.471
Renal impairment	46 (33.3%)	36 (33.6%)	82 (33.5%)	1.000
Laboratory findings				
WBC, /mm^3^	11.0 ± 10.2	8.8 ± 5.1	10.0 ± 8.4	0.032
Hemoglobin, g/dL	10.2 ± 1.8	10.4 ± 1.7	10.3 ± 1.7	0.229
Albumin, g/dL	2.8 ± 0.5	2.8 ± 0.5	2.8 ± 0.5	0.263
Total bilirubin, mg/dL	7.8 ± 9.3	7.9 ± 9.1	7.9 ± 9.2	0.929
Creatinine, mg/dL	1.7 ± 1.7	1.4 ± 1.3	1.5 ± 1.5	0.191
Na, mmol/L	131.0 ± 5.4	128.8 ± 5.8	130.0 ± 5.7	0.003
hs-CRP	75.8 ± 57.0	72.9 ± 51.0	74.6 ± 54.4	0.680
Ascitic fluid PMNLs, /mm^3^	5658.1 ± 20,215.1	2297.0 ± 3393.9	4190.2 ± 15,402.6	0.057
Ascitic fluid albumin, g/dL	0.8 ± 0.4	0.8 ± 0.5	0.8 ± 0.5	0.238
Child Pugh score	10.8 ± 1.6	10.8 ± 1.7	10.8 ± 1.6	0.933
CTP grade C (vs CTP B)	100 (72.5%)	82 (76.6%)	182 (74.3%)	0.553
MELD score	20.2 ± 9.5	18.9 ± 8.8	19.6 ± 9.2	0.253
Culture-positive SBP	36 (26.1%)	23 (21.5%)	59 (24.1%)	0.495
Antibiotic resistance	26 (72.2%)	18 (78.3%)	44 (74.6%)	0.831
Initial antibiotic therapy				
Cephalosporin	129 (93.5%)	95 (88.8%)	224 (91.4%)	0.493
Ciprofloxacin	4 (2.9%)	5 (4.7%)	9 (3.7%)	
Imipenem	5 (3.6%)	5 (4.7%)	10 (4.1%)	
Miscellaneous	0 (0.0%)	1 (0.9%)	1 (0.4%)	
Repeated paracentesis	26 (18.8%)	22 (20.6%)	48 (19.6%)	0.862
Ascitic fluid PMNLs, /mm^3^	1194.0 ± 1430.2	1477.2 ± 2453.5	1323.8 ± 1948.9	0.637
Patients with increased PMNL counts	5 (19.2%)	7 (31.8%)	12 (25.0%)	0.503
Treatment response				
Complete response	55 (39.9%)	30 (28.0%)	85 (34.7%)	0.073
Partial response	48 (34.8%)	21 (19.6%)	69 (28.2%)	0.013
Treatment failure	35 (25.4%)	56 (52.3%)	91 (37.1%)	< .001
Hospitalization (days)	25.7 ± 22.5	20.7 ± 13.6	23.6 ± 19.3	0.033

Data were expressed as number (percentage) or mean ± standard deviation.

*CA* Community acquired, *CRP* C-reactive protein, *CTP* Child Pugh, *GI* Gasointestinal, *NSBB* Non-selective beta blocker, *MELD* Model for end stage liver disease, *NC* Nosocomial, *NSBB* Non-selective beta blocker, *PMNLs* Polymorphonuclear leukocytes, *SBP* Spontaneous bacterial peritonitis, *WBC* White blood cells.

Antibiotic resistance* includes resistance to one of cefotaxime, ciprofloxacin, vancomycin, and ESBL( +).

The severity of HCC was assessed using the modified UICC staging system, in which 19 patients (17.8%) were classified as stage 1, 12 (11.2%) as stage 2, 19 (17.8%) as stage 3, and 57 (53.3%) as stage 4. According to the BCLC staging system, 4 patients (3.7%) were classified as stage 0, 11 (10.3%) as stage A, 2 (1.9%) as stage B, 18 (16.8%) as stage C, and 72 (67.3%) as stage D. Patients with HCC had higher rates of non-alcohol etiology (74.8% vs. 58.0%), previous use of antibiotics within 60 days (44.9% vs. 18.8%), and nosocomial infection (47.7% vs. 37.0%) than those without HCC. However, the CTP and MELD scores were not significantly different between the two groups.

Causative organisms were isolated from the ascitic fluid or peripheral blood in 59 cases (24.1%). Culture positivity was slightly lower among HCC patients than among non-HCC patients; however, the difference was not significant (21.5% vs. 26.1%, *P *= 0.495, Table 1). Table 2 shows the profiles of the isolated organisms. Gram-negative organisms accounted for 55.9% of all the isolated organisms (33 of 59 cases). *Escherichia coli* was the most frequently isolated organism (57.6%), followed by Enterobacter species (18.2%) and *Klebsiella pneumoniae* (12.1%). Gram-positive strains were isolated from 26 cases (44.1%). The antibiotic resistance profiles of the isolates are shown in Table 2. Among the isolated gram-negative organisms, 25 (75.8%) were resistant to ampicillin, 12 (36.4%) to ciprofloxacin, and 16 (48.5%) to cefotaxime. Furthermore, 9 of the 33 g-negative organisms also produced ESBL. Among the 26 isolated gram-positive organisms, 13 (50.0%) were resistant to ciprofloxacin, 14 (53.8%) to ampicillin, and 3 (11.5%) to vancomycin. Patients with HCC tended to have a higher rate of antibiotic resistance (78.3% vs. 72.2%) than those without HCC; however, the difference was not statistically significant.

**Table 2:** Profiles of isolated microorganisms and antibiotic resistance in patients with spontaneous bacterial peritonitis.

	Total (n = 59)	3rd cephalo-sporin	Ciprofloxacin or levofloxacin	Ampicillin or oxacillin	ESBL positive	Vancomycin
**Gram (-) organisms**	33					
* Escherichia coli*	19	10 (52.6)	9 (47.4)	12 (63.2)	8 (42.1)	–
* Klebsiella pneumoniae*	4	1 (25.0)	1 (25.0)	4 (100)	1 (25.0)	–
* Aeromonas* species	1	1 (100)	0 (0.0)	1 (100)	0 (0.0)	–
* Pseudomonas* species	3	1 (33.3)	1 (33.3)	2 (66.7)	0 (0.0)	–
* Enterobacter* species	6	3 (50.0)	1 (16.7)	6 (100)	0 (0.0)	–
**Gram (+) organisms**	26					
* Streptococcus* species	6	0 (0.0)	3 (50.0)	3 (50.0)	–	0 (0.0)
* Staphylococcus* species	10	2 (20.0)	2 (20.0)	5 (50.0)	–	0 (0.0)
* Enterococcus* species	10	3 (30.0)	8 (80.0)	6 (60.0)	–	3 (30.0)

Data were expressed as number (percentage).

*Extended spectrum β-lactamase.

### Treatment outcomes and prognosis

**Figure 1:**
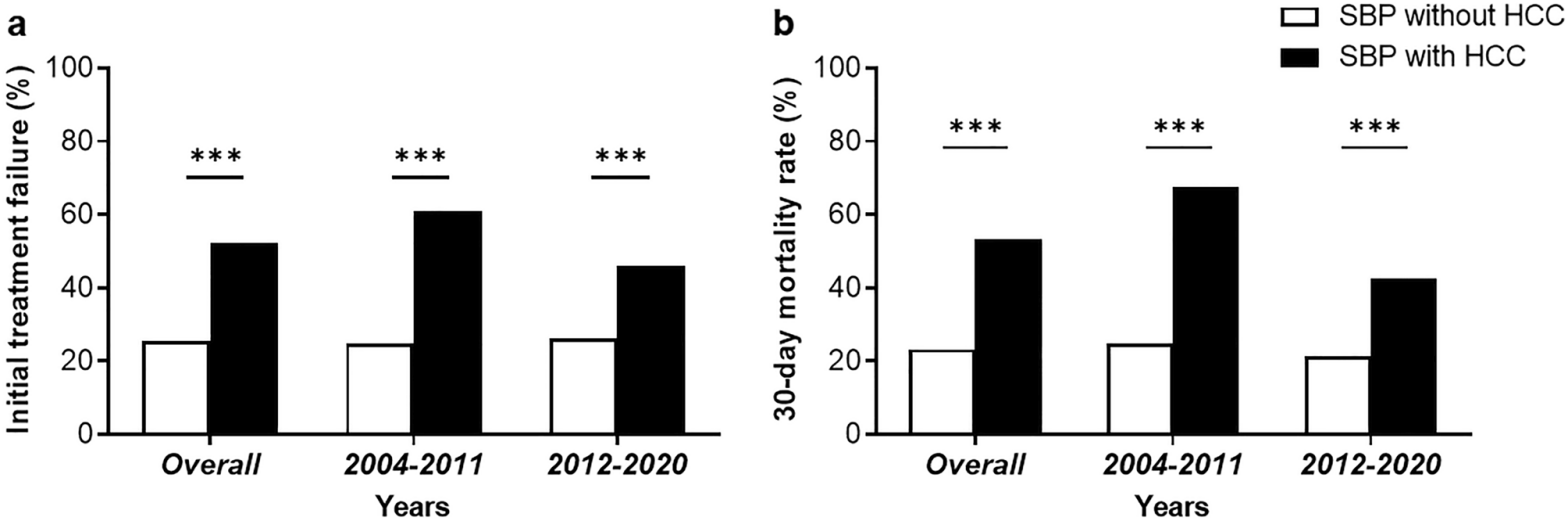
Initial treatment failure (**a**) and 30-day morality rate (**b**) in spontaneous bacterial peritonitis (SBP) patients with and without hepatocellular carcinoma (HCC).

The initial treatment response and short-term mortality rates are shown in Fig. 1. Overall, 85 cases (34.7%) of the patients with SBP showed a complete response and 69 cases (28.2%) showed a partial response in overall patients. Initial treatment failure was observed in 91 patients (37.1%). A total of 48 patients underwent repeated paracentesis for follow-up. Among them, 22 patients (20.6% of the total) had HCC, while 26 patients (18.8% of the total) had non-HCC. According to the definition, patients with worsened PMN counts during repeated paracentesis were selected as the treatment failure group, with 7 patients (31.8%) in the HCC group and 5 patients (19.2%) in the non-HCC group. Patients with SBP and HCC showed significantly higher rates of treatment failure (52.3% vs. 25.4%, *P *< 0.001) than those without non-HCC patients (Fig. 1a). Overall, the 7-day and 30-mortality were 42 cases (17.1%) and 89 cases (36.3%), respectively. The 30-day mortality rate was significantly higher in SBP patients with HCC than in those without HCC (53.3% vs. 23.2%, *P *< 0.001) (Fig. 1b). Furthermore, upon segmenting the study period into two intervals (2004–2011 and 2012–2020) for analysis, these higher rates of treatment failure and 30-day mortality among patients with both SBP and HCC were consistent across all analyzed periods.

### Factors associated with initial treatment failure

Initial treatment failure was associated with non-alcoholic etiology of cirrhosis, nosocomial infection, administration of non-selective beta-blockers (NSBB), presence of HCC, hepatic encephalopathy, renal impairment, Child–Pugh grade C, MELD score, positive ascitic fluid culture, antibiotic resistance, and initial imipenem treatment in the univariate analysis (Table 3). In the multivariate analysis, the presence of HCC (odds ratio [OR] 8.54; 95% confidence interval [CI], 1.71–58.20; *P *= 0.015), renal impairment (OR 16.37; 95% CI, 2.98–153.32; *P *= 0.004), Child–Pugh grade C (OR 29.69; 95% CI, 3.72–699.73; *P *= 0.006), and antibiotic resistance (OR 21.09; 95% CI, 2.77–301.09; *P *= 0.009) were identified as independent factors associated with initial treatment failure (Table 4).

**Table 3:**
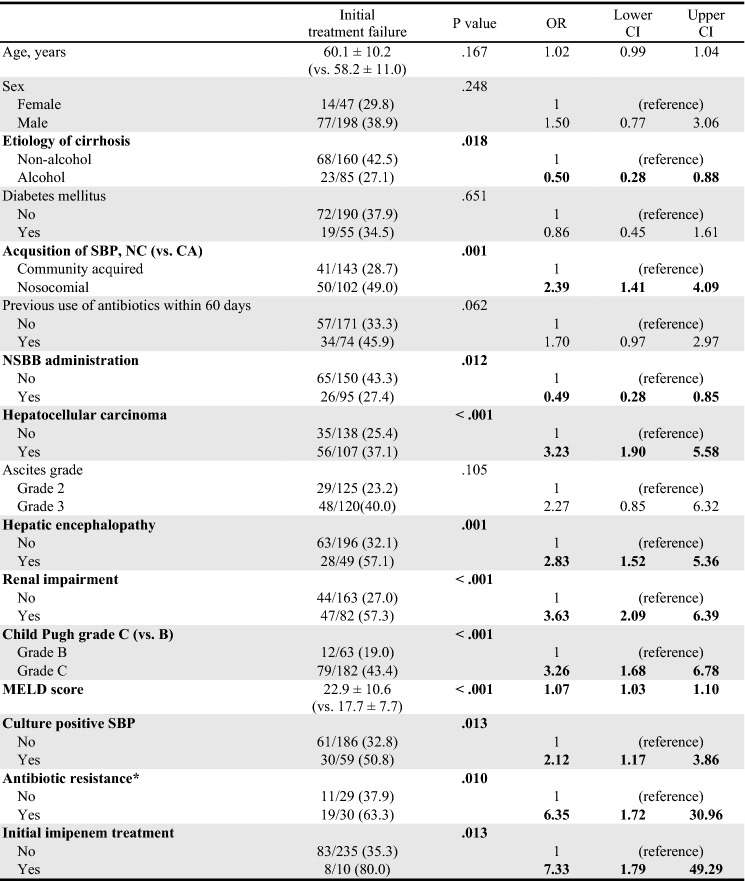
Factors associated with initial treatment failure (Univariate analysis).

*CRP* C-reactive protein, *GI* Gasointestinal, *MELD* Model for end stage liver disease, *PMNLs* Polymorphonuclear leukocytes, *SBP* Spontaneous bacterial peritonitis, *WBC* White blood cells.

Antibiotic resistance* includes resistance to one of cefotaxime, ciprofloxacin, vancomycin, and ESBL( +).

Significant values are in [bold].

**Table 4:** Factors associated with initial treatment failure (Multivariate analysis).

	*P* value	OR	Lower CI	Upper CI
**Hepatocellular carcinoma**	**0.015**	**8.54**	**1.71**	**58.20**
**Renal impairment**	**0.004**	**16.37**	**2.98**	**153.32**
**Child Pugh grade C (vs. B)**	**0.006**	**29.69**	**3.72**	**699.73**
Antibiotic resistance*	**0.009**	**21.09**	**2.77**	**301.09**

Antibiotic resistance* includes resistance to one of cefotaxime, ciprofloxacin, vancomycin, and ESBL( +).

Significant values are in [bold].

### Factors associated with Short-term(30-day) mortality

**Figure 2:**
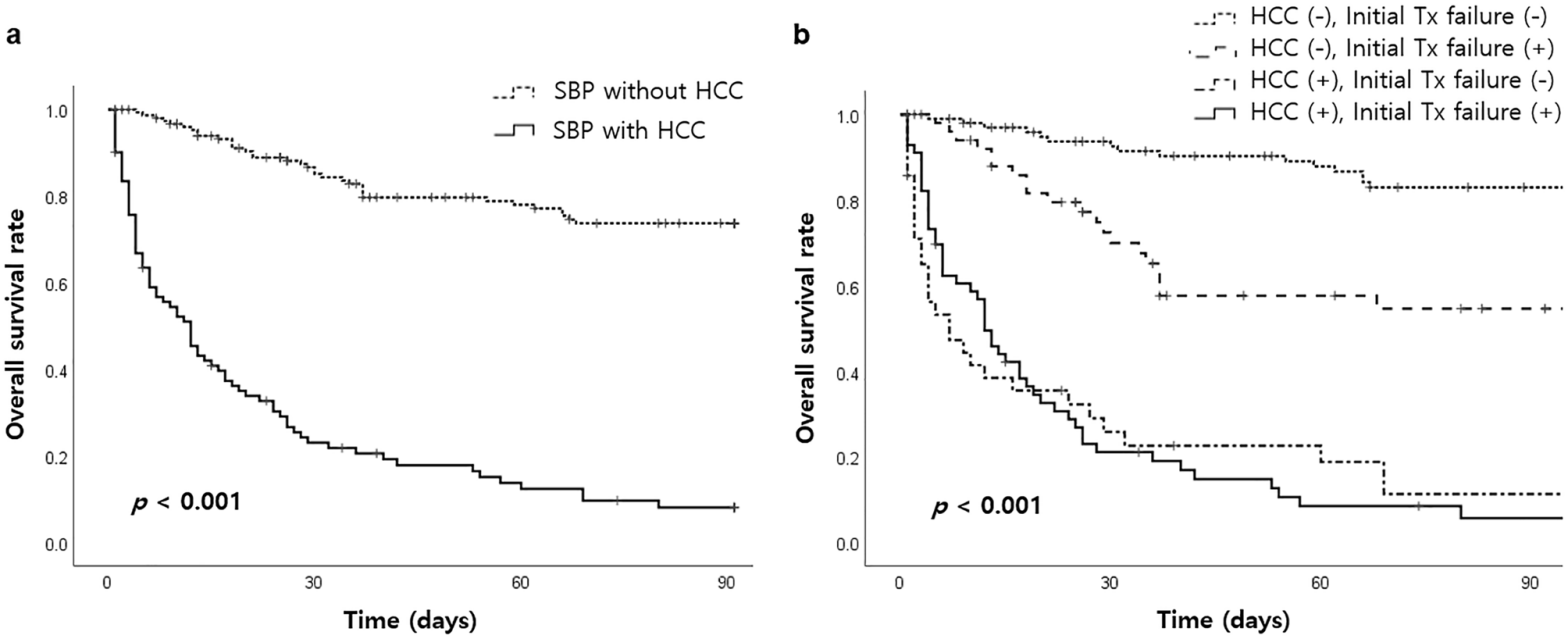
Overall survival rate according to the presence of hepatocellular carcinoma (HCC) (**a**) and subgroups based on HCC and initial treatment failure (**b**).

Short-term (30-day) mortality was associated with non-alcoholic etiology of cirrhosis, nosocomial infection, administration of NSBB, presence of HCC, hepatic encephalopathy, renal impairment, Child–Pugh grade C, MELD score, and initial treatment failure in the univariate analysis (Table 5). In the multivariate analysis, the presence of HCC (OR 3.35; 95% CI, 1.61–7.20; *P *= 0.001), hepatic encephalopathy (OR 3.45; 95% CI, 1.49–8.32; *P *= 0.005), MELD score (OR 1.05; 95% CI, 1.01–1.10; *P *= 0.015), and initial treatment failure (OR 12.23; 95% CI, 6.11–25.54; *P *< 0.001) were identified as independent factors for 30-day mortality (Table 6). Furthermore, Kaplan–Meier survival analysis showed statistically significant poor survival outcomes in patients with HCC (*P *< 0.001). Likewise, when HCC and initial treatment failure were considered as factors, they also demonstrated statistically significant associations with poor prognosis (*P *< 0.001) (Fig. 2).

**Table 5:**
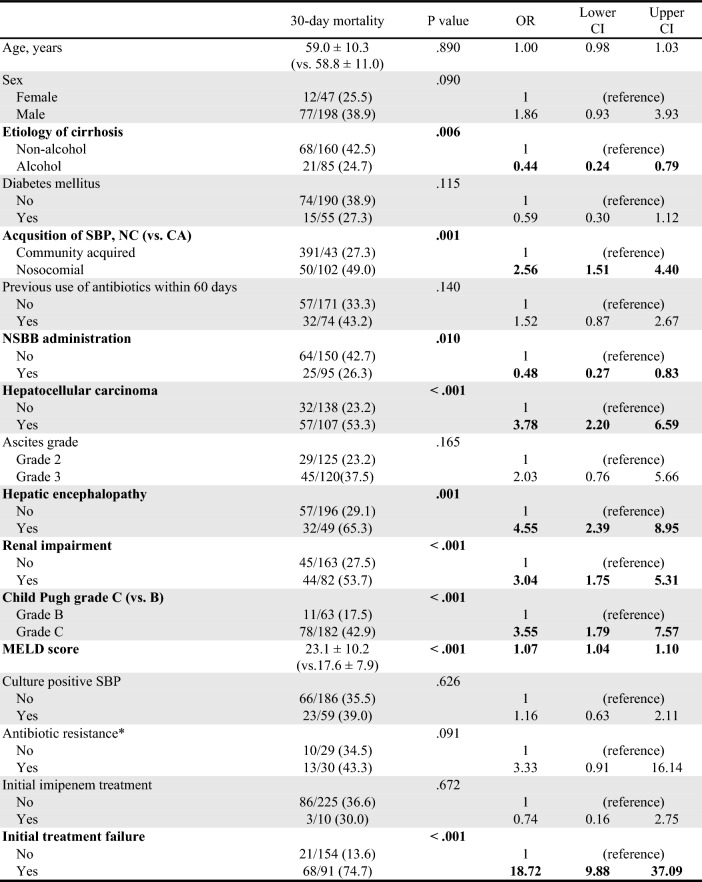
Factors associated with 30-day mortality (Univariate analysis).

*CRP* C-reactive protein, *GI* Gasointestinal, *MELD* Model for end stage liver disease, *PMNLs* Polymorphonuclear leukocytes, *SBP* Spontaneous bacterial peritonitis, *WBC* White blood cells.

Antibiotic resistance* includes resistance to one of cefotaxime, ciprofloxacin, vancomycin, and ESBL( +).

Significant values are in [bold].

**Table 6:** Factors associated with 30-day mortality (Multivariate analysis).

	*P* value	OR	Lower CI	Upper CI
**Hepatocellular carcinoma**	**0.001**	**3.35**	**1.61**	**7.20**
**Hepatic encephalopathy**	**0.005**	**3.45**	**1.49**	**8.32**
**MELD score**	**0.015**	**1.05**	**1.01**	**1.10**
**Initial treatment failure**	** < 0.001**	**12.23**	**6.11**	**25.54**

Significant values are in [bold].

## Discussion

Despite the management of SBP based on current practice guidelines recommending empirical antibiotic therapy after the diagnosis of SBP without the result of microbiological culture, treatment failure and mortality related to bacterial infection are still substantially high. Analysis of the present study revealed high rates of treatment failure and short-term mortality, even in recent data from 2012 to 2020. These findings highlight SBP as a major challenge in the management of cirrhotic patients and indicate the need for further studies to identify more effective strategies for improving outcomes in this serious complication.

The causative strains of SBP and the rates of antibiotic resistance have been altered. Previous studies reported that culture of microorganisms was positive in up to 40% of SBP patients with most common pathogens, including gram-negative bacteria, usually *Escherichia coli*. Thus, third-generation cephalosporins are commonly recommended as initial antibiotic therapy^1,15^. In this study, over than 90% of patients were also initially treated with third generation cephalosporins. However, SBP caused by third-generation cephalosporin-resistant bacteria has been implicated in treatment failure. A previous multicenter study of Korean cirrhotic patients with SBP conducted in 2009 reported that the prevalence of gram-negative bacilli resistant to cefotaxime was 17%^16^, while it was substantially increased to 52.6% in the present study. Furthermore, the prevalence of gram-positive bacteria, such as Streptococcus and Enterococcus, increased to 44% compared to 17% in a previous study^16^.

The response to antibiotic treatment is one of the most important factors in SBP treatment. The treatment response or failure reflects the overall prognosis of patients in clinical practice. In this study, initial treatment failure was a significant factor related to 30-day mortality, and factors related to initial treatment failure were HCC, renal impairment, CTP score, and antibiotic resistance. Patients with cirrhosis who develop SBP have been reported to experience a high incidence of renal dysfunction. One-third of patients with SBP develop renal failure despite successful treatment of infection, which is a major contributor to high mortality. A recent systematic review has identified renal dysfunction as the most important independent predictor of mortality. In our study, the frequency of renal impairment at diagnosis was 33.5%, and the serum creatinine level at the time of SBP diagnosis was statistically significant in initial treatment failure. With regard to antibiotic resistance, the causative organism of SBP has changed, with a shift towards gram-positive infections and increased multidrug-resistant bacteria, such as extended-spectrum β-lactamase-producing Enterobacteriaceae^14,17^. In circumstances where more than 90% of 3rd cephalosporin was administered as an initial treatment in this study, antibiotics resistance may be the cause of initial treatment failure.

Multiple studies have proposed multiple predictors of poor prognosis in patients with SBP. Predictors of poor prognosis in SBP include old age, higher Child–Pugh score, nosocomial origin, encephalopathy, elevated serum creatinine and bilirubin levels, ascites culture positivity, presence of bacteremia, and infection with resistant organisms^7^. Most of the risk factors are related to liver function and systemic conditions. Previous studies have revealed that a high Child–Pugh score or MELD score is a predictor of mortality in SBP^18–24^. Tandon et al. highlighted the MELD score as the main prognostic factor of mortality in cirrhotic patients with SBP and renal dysfunction. In this study, the MELD score, hepatic encephalopathy, and initial treatment failure were independent factors associated with 30-day mortality in patients with SBP.

The most important finding in this study was that HCC patients showed significantly higher treatment failure rates than non-HCC patients with SBP, and HCC was significantly associated with 30-day mortality in SBP patients. Moreover, this significant difference was consistently observed even upon segmenting the entire study period for analysis. A prospective Greek multicenter study reported that a history of HCC is associated with overall survival along with MELD score, lactate, albumin, and treatment with vasopressors^12^. Another study reported that HCC is associated with higher mortality rates in cirrhotic patients with SBP^11^. Moreover Tu et al. established a multivariate diagnostic model for asymptomatic SBP in cirrhotic patients with ascites based on the blood neutrophil percentage, HCC, MELD, PMN, and renal dysfunction^13^. However, Kim JH et al., in a retrospective study including 123 patients comparing prognosis of SBP between the HCC and non-HCC groups, exceptionally demonstrated that the prognosis of the HCC group was relatively less severe than that of the non-HCC group because of the lower antibiotic resistance rate in the HCC group^10^. We tried to determine the different clinical factors between the two groups; SBP patients with HCC showed higher rates of non-alcohol etiology (74.8% vs. 58.0%) and previous use of antibiotics within 60 days (44.9% vs. 18.8%) than those without HCC. However, the CTP and MELD scores were not significantly different between the two groups. It is possible to infer that HCC patients have more opportunities to be exposed to antibiotics, such as third-generation cephalosporins or quinolones, because of frequent hospitalization, which might be related to antibiotic resistance. In addition, repeated locoregional or systemic anticancer treatments may further aggravate the impairment of the host immune system. Thus, all of these clinical factors are potentially associated with an increased risk of initial treatment failure and short-term mortality for SBP in cirrhotic patients with HCC.

The present study had several limitations. First, the isolation rate of microorganisms was lower than that reported in previous studies reporting 39–41%. A low culture-positive rate can reduce the accuracy of the isolated microorganism distribution and antibiotic resistance analysis results. Second, this was a single-center retrospective study. Further large-scale, multicenter prospective studies are essential to confirm our proposal.

In conclusion, this study showed that HCC is an independent risk factor for initial treatment failure and high short-term mortality in patients with cirrhosis with SBP. Thus, more aggressive treatment strategies with broad-spectrum antibiotics are needed in the early stages of SBP treatment to reduce initial treatment and eventually mortality in cirrhotic patients with HCC.

## Materials and methods

### Study populations and definitions

This study retrospectively enrolled cirrhotic patients with ascites who were diagnosed with SBP by diagnostic or routine paracentesis at Jeonbuk National University Hospital between 2004 and 2020. Patients who were diagnosed with SBP multiple times during the study period were only included based on their first occurrence. Liver cirrhosis was diagnosed based on clinical evidence of portal hypertension manifesting as thrombocytopenia (< 100,000/μl), splenomegaly, ascites, varices, or hepatic encephalopathy with compatible findings on radiologic imaging^15^. The diagnosis of HCC was confirmed by liver biopsy or typical imaging features of hepatic nodules more than 1 cm (i.e., arterial phase hyperenhancement with washout appearance in the portal venous, delayed, or hepatobiliary phases) on dynamic computed tomography (CT), dynamic magnetic resonance imaging (MRI), or MRI using a hepatocyte-specific contrast agent^25^. The diagnosis of SBP was based on polymorphonuclear leukocytes (PMNs) ≥ 250 cells/mm^3^ in ascitic fluid without evident intra-abdominal infection^15^. In this study, pathogen identification was not considered essential for the diagnosis of SBP. We excluded patients with non-neutrocytic bacteremia (< 250 cells/mm^3^ PMNs in ascitic fluid), polymicrobial infection, and cases of secondary peritonitis. Other exclusion criteria were positivity for human immunodeficiency virus infection, heart failure, and organic nephropathy (proteinuria, hematuria, or abnormal findings on renal ultrasonography).

We reviewed the medical records and laboratory databases of all the study patients. In this study, we compared the clinical features and treatment outcomes between patients with SBP with and without HCC. The clinical presentation and laboratory data for every SBP episode were gathered. Information regarding the etiology of liver cirrhosis and comorbid medical conditions was collected. We investigated whether the patient had taken oral quinolones within 60 days before the SBP episode. SBP was classified as ‘community-acquired’ if the infection was diagnosed within first 48 h of hospitalization, and ‘nosocomial’ if the infections diagnosed after more than 48 h of hospitalization. The Child–Pugh score were calculated to assess the grade of liver cirrhosis at the time of SBP diagnosis. Renal impairment at SBP diagnosis was defined as elevation of serum creatinine greater than 1.5 mg/dL^16^. This study was conducted in compliance with the World Medical Association Declaration of Helsinki and approved by the ethics committee of Jeonbuk National University Hospital. Jeonbuk National University Hospital Institutional Review Board (IRB) has waived informed consent for this study.

### Clinical treatment protocol and assessment of treatment outcomes

In practice, blood and/or ascitic fluid was collected from each patient and inoculated into blood culture bottles before the initiation of treatment. The patient was empirically treated with intravenous third-generation cephalosporin. It was subsequently maintained or escalated depending on the clinical course and antibiotic sensitivity of isolated microorganisms, up to a planned period of 5–10 days during hospitalization^26^. At the time of the SBP diagnosis, intravenous albumin 20–40 g daily was administered with antibiotic therapy.

Blood and ascitic fluid samples were injected into BacT culture media bottles (Bactec NR-860 system, Johnson Laboratories, Towson, MD, USA) and cultured at 35 °C for 7 days. Microorganisms and their antibiotic susceptibilities were identified using a MicroScan system (Dade Behring, West Sacramento, CA, USA). Extended spectrum beta-lactamase (ESBL) production was detected using the double-disc synergy test and E-test method (AB Biodisk, Solna, Sweden)^27^. For the purposes of this study, Gram-negative bacilli with intermediate in vitro susceptibility to third generation cephalosporins, penicillines, quinolones, or ESBL-producing organisms were considered resistant to these antibiotics.

Treatment outcomes were assessed based on the initial treatment response and short-term (7-day and 30-day) mortality rates. In this study, the initial treatment response was evaluated 72 h after the onset of antimicrobial therapy and was classified as follows: 'complete response' for patients who had resolution of fever, leukocytosis and all signs of infection; 'partial response' for patients who had abatement of abnormalities in the above parameters without complete resolution; 'failure' for patients who had died or experienced worsening of clinical signs and symptoms of infection and/or had increased neutrophil count in ascitic fluid compared to levels at the time of diagnosis^5^. We compared the initial treatment response and short-term mortality of patients with cirrhosis with SBP between the non-HCC and HCC groups which were further examined by stratifying them into distinct time periods. We also analyzed the clinical risk factors associated with initial treatment failure and short-term mortality.

### Statistical analysis

The results are reported as a number (percentage) or mean ± standard deviation. Continuous variables were compared using the 2-tailed student’s *t*-test, and categorical data using the chi-square test or Fisher’s exact test. Factors associated with initial treatment failure and 30-day mortality were analyzed using multivariate analysis with a logistic regression model. Statistical significance was set at *P *< 0.05. Data were transferred into a Microsoft EXCEL database (Microsoft Office 365; Microsoft Corporation, Redmond, WA, USA), and analyzed using IBM SPSS Statistics for Windows (version 25.0; IBM Corporation, Armonk, NY, USA).

## Data Availability

The datasets used and analyzed during the current study are available from the corresponding author on reasonable request.
